# Traumatic Tetanus

**Published:** 1877-05

**Authors:** Ezra Read

**Affiliations:** Terre Haute, Ind.


### TRAUMATIC TETANUS.

By EZRA READ, M. D., of Terre Haute, Ind.

[Read before the Indiana State Medical Society.]

Throughout my professional life, since by study, reflection and experience I had carefully settled the question in my own mind, I have declared in public and in private circles, that *traumatic tetanus* was incurable by any of the remedies now known.

I made this declaration before this learned association at its last meeting; but as Solon said that he learned something in his old age, every day, and continually advanced in the paths of knowledge, I trust it may not be deemed affectation to assert that I have, in the last four months, learned at least one fact, that traumatic tetanus is curable. Prior to this time, in nearly half a hundred cases within my own observation, every one terminated in death, and I came to distrust reports and written opinions to the contrary.

As this disease has existed from the beginning of mankind upon earth, and will to the end, it may not be unbecoming or uninstructive to render to those I have the honor of addressing, a brief collection of the opinions upon the subject of some of the eminent physicians who have preceded us; especially as I am incapable of advancing those of my own upon a matter so unsettled by the profession.

No writer upon this disease, known to me, is earlier than Hippocrates, and, for all practical purposes, that is far enough back. In his aphorism relating to this, he says: "Spasm supervening upon a wound is fatal," and in another: "Such persons as are seized with tetanus die within few days, or, if they pass these, they recover." His method of cure was venesection, strong Cretan wine, bathing the legs and feet in hot water, and the arms, and hands, the spinal column in its entire length from neck to sacrum to be wrapped in skin smeared with wax. Intervals were used for the application of hot fomentations, by means of leather bottles filled with hot water, and covering up in the bed.

He recommended the use of the bryony, in fragrant wine, and carrot, to be given to the patient early in the morning, fasting. Prior to and succeeding this, warm barley gruel was to be used and diluted wine.

The bryony is said to be a thorough and hydragogue cathartic, and strongly diuretic.

He concludes by saying, "If the disease yield to these means, so much the better, but, if otherwise, you must prognosticate accordingly." I do not know that it may be regarded an unfavorable reflection upon the profession to say that this method of cure has not been much improved since the above was written.

Celsus coincided with Hippocrates in relation to the danger of this disease, and that it usually proved fatal before the fourth day. Living beyond that time was favorable to recovery. He differs with Asclepiades, who advocated bleeding, but recommended scarification and cupping upon the neck, and burning it with the actual cautery, or corroding it with mustard.

He gave castor, and pepper and clysters, and applied hot fomentations of water to the neck, and also anointed these parts with liquid cerate, and then applied ox bladders, or bottles, filled with hot oil, or a hot meal poultice, or round pepper bruised with figs, also a fomentation with moist salt.

He recommended the neck, shoulders and spine to be thoroughly rubbed with old oil, the hair to be closely cut, and the head moistened with hot orris oil, or that of cypress, and to be covered with a cap.

Friction, though serviceable to all the vertebrae of the body, is particularly so to those of the neck, and should be resorted to *die nocteque.*

Food, requiring mastication, should be for a long time avoided; gruel, soft and fresh boiled eggs are to be used, and at later convalescence, wine. All cold should especially be avoided, and warmth carefully preserved.

These eminent physicians will thus be observed to have had corresponding views upon this disease, and did not widely differ from all since.

Paulus Aegineta recites, among other symptoms, a moaning respiration, pulse small, red face, eyes protruded, and sometimes a sardonic laugh, with scanty urine, and occasionally singultus. He regards the supervention of fever to be favorable, as did Hippocrates, Galen and Avicenna. The treatment recommended is very similar to that of the writers already quoted. Hot applications to the neck, bleeding, hot oil bath, cupping, musk, assafœtida and gum ammoniac.

He agrees with Hippocrates that cold water affusions are hazardous remedies. This was recommended by Dr. Currie, of Liverpool, but abandoned, and was not adopted by the profession.

Aetius, Aribasius and their cotemporaries, recommended bleeding, hot fomentations, and the oil bath, made by adding one-fifth oil to water. Arctæus describes tetanus to be painful, sometimes proving speedily fatal and *always difficult to cure.* His treatment was soothing and relaxant, with hot applications, a warm, soft bed, bleeding, cupping and suppurative applications to the wound. Cadius Aurelianus, Haratianus and others of this age, adopted this method.

The Arabian physicians, Avicenna, Albucasis and Rhazes adopted the Greek method, and, in fact, the whole medical world since. The disease has been obscure, with like treatment.

I come now to the modern views of surgeons upon this disease, its derivation being from the Greek word *teiro*, to stretch, implying that condition of the muscles of the spine, their terminal tendons, either drawn forward, emprosthotonos, or backwards, opisthotonos. It is an involuntary spasmodic action of the muscles of the spinal column, from a punctum saliens, inducing such condition through nervous action.

Tetanus is usually of gradual invasion, but a case is recorded by Professor Robison, of Edinburgh, of a negro, who had wounded his thumb, from which he died of tetanus in fifteen minutes, but this seems so unreasonable that it is unworthy of credit, noted as it is by a gentleman so respected for his professional attainments.

Stiffness about the neck, which increases to the extent of rendering the motion of the head difficult and painful; an uneasiness felt at the root of the tongue, rendering mastication of food, or swallowing of liquids painful or impossible, causing convulsive action, and the disinclination to make the attempt, are prominent and alarming symptoms. There is severe pain at the bottom of the sternum, which darts from this point backwards in the direction of the spine. Violent spasms seize all the muscles of the neck, and the head is drawn forwards or backwards, in obedience to the greater or lesser action of the flexor or extensor muscles. The inferior maxilla is drawn more rigidly to the superior, and the jaws are fixed, are locked. It is the characteristic and unmistakable symptom of the disease, its pathognomonic culmination. This spasmodic condition returns at short intervals, induced by the least possible excitement, as attempting to swallow, or being touched, or moved, or quickly spoken to, and always attended with most excruciating pain. The head is incapable of lateral turning, the arms are rigid and fixed, the legs are rigid. The abdominal muscles are so rigidly contracted that the belly feels hard and tense; the tongue loses its volition, and is spasmodically thrust forwards between the teeth, to be torn and lacerated by violent spasms, unless properly protected. In its severe form, the muscles of the face are contracted, the forehead is drawn into furrows, the eyes distorted, fixed and motionless, the nose is drawn up, the cheeks are retracted towards the ears, and the whole expression is painfully changed, and not unfrequently there is a wild, sardonic grin upon the face.

There is little or no fever; the surface is usually bathed with sweat, especially about the face and forehead; the pulse is hurried and small, generally above one hundred; the secretions are but little changed. Sleep almost entirely forsakes the patient, from convulsive interruptions, and the whole condition is that productive of pity and pain to physician and attendants.

The modern writers who have had the largest experience in this disease, concur in the opinion of its imminent danger, and the general methods to be observed in its treatment. Sir James McGregor, the Chief British Army Surgeon in Spain and Portugal, and Baron Larrey, both had large experience in hot climates, and from gunshot wounds. The former, in his report, says it occurred in every description and stage of wounds from the slightest to the most formidable; it followed the healthy and the sloughing, the incised and lacerated, the the most simple and the most complicated, and occurred at uncertain periods, but after twenty-two days, but little apprehension was to be had. It attacks all ages and conditions, but infants and the middle-aged are most liable. The feeble and weak are said to be less liable, and the female than the male.

Although a disease of all climates and all seasons, it is most common in marshy districts, bordering upon the sea, and in the hot months of hot climates, where it is an ordinary consequence of all wounds. Cold and moisture are recognized agents in inducing the disease, after wounds, and especially when applied to the surface in a heated condition. All atmospheric vicissitudes are aiding conditions.

In our present state of knowledge, we cannot indulge the hope of much success in the treatment of this disease. All remedies have been tried and with limited success.

The knife itself reveals no lesion after death. A little redness of the mucous surface of the œsophagus and the cardiac region of the stomach alone exhibits increased vascularity, and slight vascular inflammation, with contraction of the pharynx and œsophagus. In fact, no injury of any part or organ develops sufficient cause of death.

It must, therefore, be sought for in the nervous system, and the result of the shock to this tissue, which checks the machinery of life without a morbid trace to direct the mind to the invaded part.

Larrey, McGregor, and others scarcely less eminent, have resorted to amputation repeatedly, with the hope of relief, but in not one single instance was it of any use whatever. The division of the nerve has, in like manner, been made, with like results. As the wound is generally healed before the spasm sets in, or, if open, the suppuration diminishes, irritating substances have been applied to restore the inflamed condition of the wound, and it would seem a most reasonable means.

Doctors Rush and Hosack advocated the use of stimulants, and especially of wine. Barks and wine have been used; calomel, and all antispasmodic remedies,—blisters, cupping, tobacco clysters, the cold and hot baths, and, in fact, every remedy which could suggest itself to anxious and inquiring minds.

In Jamaica, and other West India Islands, the cold bath and opium have been favorably mentioned. Dr. Drummond, of the former island, says, "I am of opinion that opium and cold baths will answer every intention in tetanus and such like diseases, for while the opium diminishes the irritability and gives a truce from the violent symptoms, the cold bath produces that wonderful tonic effect so observable in this and some other cases." "Perhaps bark and wine joined with these would render the cure more certain." The method of using the cold bath was to plunge the patient in cold water or the sea, wipe dry, put to bed, and administer twenty or thirty drops of laudanum.

Opposed to this, by eminent British surgeons serving in Spain, is the opinion that they had found the "cold bath worse than useless."

Baron Larrey killed one patient with the cold bath, and it stands condemned by Hippocrates, Cullen, Callison, and the majority of intelligent surgeons in every age. Its utility, I think, is doubtful in all convulsive diseases, and I am not sure but that my friend, Dr. Swafford, and myself, in our army practice, hastened the death of a poor soldier in Alabama, by means of a cold shower bath. In addition to the internal use of mercury, even to salivation, the mercurial friction has been by some recommended, but has found *its* objectors in the cases of Drs. Guthrie and Emery, army surgeons, who used it in unlimited quantities, without any success whatever.

In speaking of Dr. Rush's views of stimulants, I should have mentioned his opinion to have been, that it is a disease essentially connected with debility, and he recommends most powerful stimulants of brandy, wine, ether, preparations of ammonia, bark and cordials, with dilatation of the wound, and a dressing of oil of turpentine to it. The use of Prussic acid has been repeated with favorable results. Blistering along the entire spinal column has been extensively and successfully used, and is, perhaps, the best known external application. It commends itself, I think, to the approbation of intelligent physicians everywhere, and has at least some reason and philosophy in its *methodus medendi.* Having presented the prominently received opinions of the medical world, I now present the following case, but just recovered.

Michael N., a native of Terre Haute, of Irish parentage, well grown and healthy, and a machinist by trade, was at work in one of the shops in St. Louis, and while holding a piece of railroad iron in the act of being cut, a fragment was thrown off, passing through the muscles, filling the space between the thumb and forefinger. The laceration was slight, with but little pain. He consulted a physician, who applied the tr. of arnica, and in six days it was completely healed. He returned to his home in this city, and, being upon the street on the evening of the 25th of August, (he was wounded the 16th of August) and creating more noise than the law allows at a political gathering, he was arrested and confined in the station-house four days. He had two chills, while confined, and the room being damp and chilly, he was released, and on the 31st of August, fifteen days from date of injury, he was seized with tetanic spasms and stiffened jaws.

I was immediately sent for, and with a stiffened and convulsed patient before me, and a small army lying quietly under the earth from similar condition, I was not much encouraged as to future results, and so expressed myself to the family.

His tongue was already wounded by protrusion, although the convulsions had not reached to the extent which followed.

As presented, he was lying upon his back with a ghastly expression of countenance, his jaws firmly fixed, and all the cervical muscles rigid and hard. His hands were folded across the breast, and immovable. His pulse was 112 and small; surface cool and moist; had no pain; bowels and urinary organs in healthy condition; had not slept previous night, and seemed and expressed himself to be comfortable in the absence of the spasms, which came on at irregular intervals, from 20 to 40 minutes. His head was constantly drawn backwards, but increased during spasmodic excitement.

He was particularly sensitive to noise and the touch, which invariably excited to spasmodic action. All of the above recited symptoms gradually increased, with the exception of frequency of pulse, which remained the same, varying from 100 to 110—for three days, when the disease seemed to have reached to its fullness, and from that time to resolution, there was scarcely an appreciable change. Reaching its acme, he had pain between the shoulders, and in the neck, loins, and scrobiculus cordis, and extending to the spine in the course of the diaphragm, with a tense and hard belly.

On the fourth day he had the sardonic grin, with retracted cheeks, and forehead drawn into furrows, eyes distorted and fixed, nose drawn up, and a most painful expression of countenance.

In this condition he remained without any fear whatever for thirty days, when the convulsions gradually ceased with a general relaxation of all the muscles which had so long been imprisoned in their rigid cells. During all of this time he and his family declared that he had no sleep; but this, of course, should be received *cum grano salis.*

I can readily understand that the sleep was limited from convulsive interruption, but not that there was a total absence of it. This, like the daily condition, forbids the recital of daily symptoms, and I shall not weary your patience with further details.

The treatment consisted in the administration of 3 grains of calomel daily in 1 grain doses, for five days, with 20 grs. of quinine in 5 gr. doses. This was directed more against the malarial influence, than with the hope of influencing the disease except indirectly by removing this all-pervading poison, which to a greater or lesser extent impresses all other diseases and conditions.

I gave hydrate of chloral for three days, which lessened the frequency of the spasms, but apprehending its cumulative tendency, I dispensed with it and substituted whisky, and of this he drank three gallons a week, or a little more than three pints daily. He called for it constantly, and was assured in his own mind that this was the very remedy to cure him. I felt myself a greater reliance upon it than any other remedy suggested to my mind, and I am now fully persuaded that the whisky brought about the cure. He was really drunk from day to day, but, like those without disease in like condition, he was happy, and immediately following a convulsive struggle would resume his cheerfulness and strong hope of recovery. During all of his illness I gave him animal broths, beef tea, and corn-meal gruel.

He is now walking around apparently well, but very stiff in the loins and hips.

He has the movement of an old man with rheumatism in the back. He was in my office four days ago, having walked half a mile, and is well, except the stiffness just mentioned.

This is the first case of tetanus that I have ever seen cured, but in the future, and as long as our good sister State of Kentucky continues to manufacture its copper distilled whisky, I shall not pronounce tetanus a mortal and incurable disease.

